# Supplementary figures and images for: Agro-morphological and genetic variability analysis in oat germplasms with special emphasis on food and feed
Source: PLoS One. 2023 Feb 8;18(2):e0280450. doi: 10.1371/journal.pone.0280450 (PMC9907803; doi:10.1371/journal.pone.0280450)

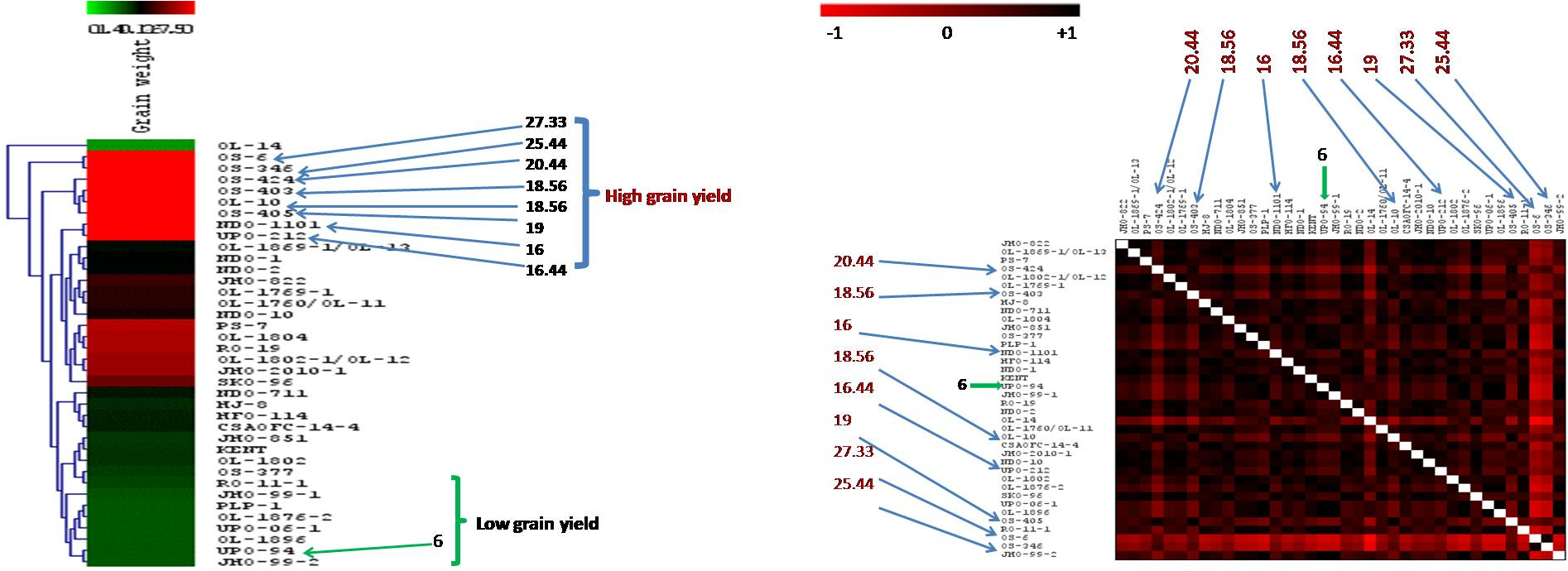

Supplement: S1 Fig — (TIF) [file pone.0280450.s001.tif]

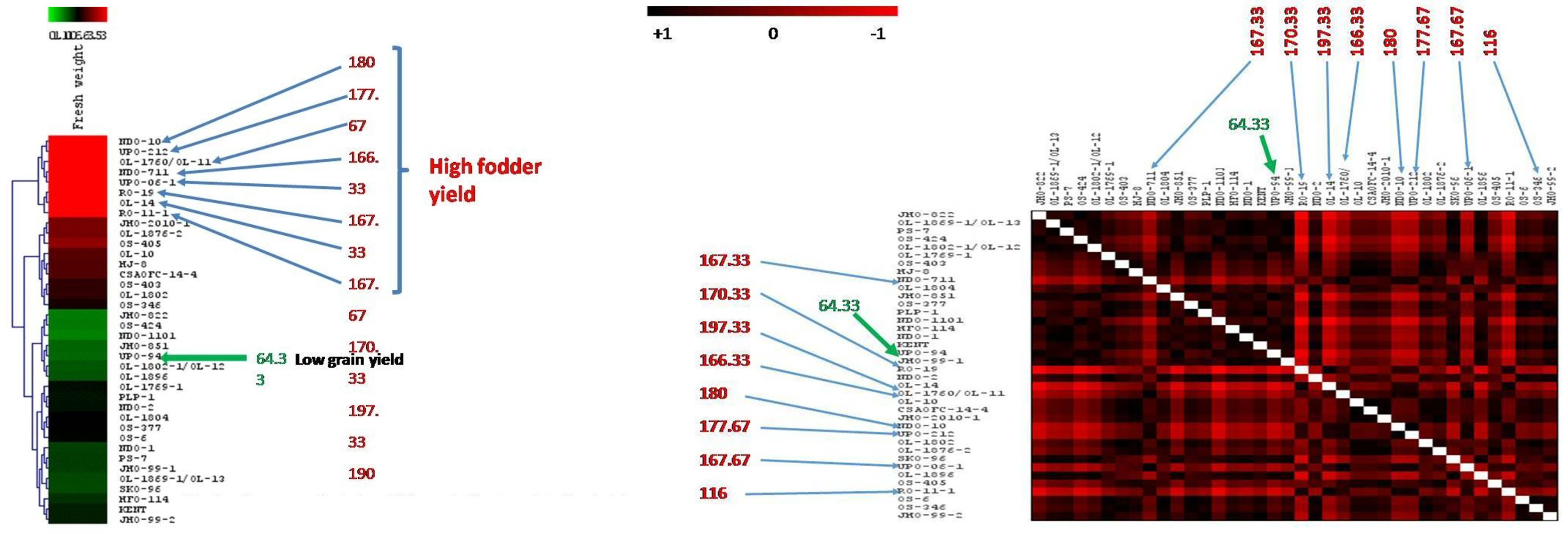

Supplement: S2 Fig — (TIF) [file pone.0280450.s002.tif]
