# Supplementary material for: Agro-morphological and genetic variability analysis in oat germplasms with special emphasis on food and feed
Source: PLoS One. 2023 Feb 8;18(2):e0280450. doi: 10.1371/journal.pone.0280450 (PMC9907803; doi:10.1371/journal.pone.0280450)
Supplement: S1 Table — (DOCX) [file pone.0280450.s003.docx]

| S. No | Genotypes | Fresh Weight (g) | Grain Weight (g) | Source |
| --- | --- | --- | --- | --- |
| 1 | JHO-822 | 56.33 | 16.44 | IGFRI Jhansi |
| 2 | OL-1869-1/OL-13 | 80 | 13.67 | IGFRI Jhansi |
| 3 | PS-7 | 84.67 | 15.50 | IGFRI Jhansi |
| 4 | OS-424 | 58.33 | 20.44 | IGFRI Jhansi |
| 5 | OL-1802-1/OL-12 | 73.33 | 15.333 | IGFRI Jhansi |
| 6 | OL-1769-1 | 104.33 | 14.56 | IGFRI Jhansi |
| 7 | OS-403 | 121. | 18.56 | IGFRI Jhansi |
| 8 | HJ-8 | 131.67 | 12 | IGFRI Jhansi |
| 9 | NDO-711 | 167.33 | 13 | IGFRI Jhansi |
| 10 | OL-1804 | 110. | 15.44 | IGFRI Jhansi |
| 11 | JHO-851 | 67 | 11.33 | IGFRI Jhansi |
| 12 | OS-377 | 110.67 | 11 | IGFRI Jhansi |
| 13 | PLP-1 | 102. | 9.44 | IGFRI Jhansi |
| 14 | NDO-1101 | 54.33 | 16 | IGFRI Jhansi |
| 15 | HFO-114 | 89.33 | 12.44 | IGFRI Jhansi |
| 16 | NDO-1 | 82.67 | 14.11 | IGFRI Jhansi |
| 17 | KENT | 97 | 11.44 | IGFRI Jhansi |
| 18 | UPO-94 | 65.33 | 9.56 | IGFRI Jhansi |
| 19 | JHO-99-1 | 84.33 | 9.78 | IGFRI Jhansi |
| 20 | RO-19 | 170.33 | 15.44 | IGFRI Jhansi |
| 21 | NDO-2 | 103. | 14 | IGFRI Jhansi |
| 22 | OL-14 | 197.33 | 6 | IGFRI Jhansi |
| 23 | OL-1760/OL-11 | 166.33 | 14.56 | IGFRI Jhansi |
| 24 | OL-10 | 129.67 | 18.56 | IGFRI Jhansi |
| 25 | CSAOFC-14-4 | 128.67 | 12.56 | IGFRI Jhansi |
| 26 | JHO-2010-1 | 137.33 | 15.33 | IGFRI Jhansi |
| 27 | NDO-10 | 180 | 14.44 | IGFRI Jhansi |
| 28 | UPO-212 | 177.67 | 16.44 | IGFRI Jhansi |
| 29 | OL-1802 | 121.33 | 11.56 | IGFRI Jhansi |
| 30 | OL-1876-2 | 136.67 | 9.44 | IGFRI Jhansi |
| 31 | SKO-96 | 79 | 15 | IGFRI Jhansi |
| 32 | UPO-06-1 | 167.67 | 9.44 | IGFRI Jhansi |
| 33 | OL-1896 | 73.33 | 9.44 | IGFRI Jhansi |
| 34 | OS-405 | 142.33 | 19 | IGFRI Jhansi |
| 35 | RO-11-1 | 190 | 10.56 | IGFRI Jhansi |
| 36 | OS-6 | 108.33 | 27.33 | IGFRI Jhansi |
| 37 | OS-346 | 116 | 25.44 | IGFRI Jhansi |
| 38 | JHO-99-2 | 97 | 9.50 | IGFRI Jhansi |

Supplementary Table 1-Description of the 38 germplasms used for the identification of high grain yielding and fresh weight (for fodder purpose) germplasms of oats
