# Supplementary material for: Agro-morphological and genetic variability analysis in oat germplasms with special emphasis on food and feed
Source: PLoS One. 2023 Feb 8;18(2):e0280450. doi: 10.1371/journal.pone.0280450 (PMC9907803; doi:10.1371/journal.pone.0280450)
Supplement: S2 Table — (DOCX) [file pone.0280450.s004.docx]

Supplementary Table 2-Performance of oat genotypes for 12 characters along with phenotypic and genotypic data analysis using statistical tools

| S. No | Genotypes | Plant Height(cm) | Tillers per Plant | Leaves per Plant | Leaf Length( cm) | Leaf Width(cm) | Fresh Weight (g) | Leaf Area(cm^2^) | Leaf Area Index | Dry Weight (g.) | Harvest index (g) | Days of 50% Flowering | Grain Weight (g) |
| --- | --- | --- | --- | --- | --- | --- | --- | --- | --- | --- | --- | --- | --- |
| 1 | JHO-822 | 75 | 6.67 | 20.33 | 31 | 1.30 | 56.33 | 631 | 1.43 | 31.63 | 34.24 | 85 | 16.44 |
| 2 | OL-1869-1/OL-13 | 83.67 | 8 | 26.33 | 31.33 | 1.70 | 80 | 1056 | 2.34 | 23.69 | 37.17 | 91.33 | 13.67 |
| 3 | PS-7 | 95 | 7.67 | 30 | 34 | 1.60 | 84.67 | 1094.33 | 3.09 | 36 | 31.37 | 80 | 15.50 |
| 4 | OS-424 | 92.67 | 3.67 | 25 | 38 | 2.00 | 58.33 | 1306.33 | 2.82 | 36.56 | 36.31 | 88 | 20.44 |
| 5 | OL-1802-1/OL-12 | 73.33 | 4 | 22.67 | 45 | 1.53 | 73.33 | 1201.67 | 2.66 | 37 | 28.85 | 86 | 15.333 |
| 6 | OL-1769-1 | 98.33 | 6 | 28 | 36 | 1.37 | 104.33 | 1046. | 2.32 | 32.11 | 31.21 | 92 | 14.56 |
| 7 | OS-403 | 109.33 | 7 | 30 | 40 | 1.87 | 121. | 1606.67 | 3.57 | 44.56 | 29.48 | 86 | 18.56 |
| 8 | HJ-8 | 110.67 | 6.67 | 31.33 | 45 | 2.10 | 131.67 | 2412 | 5.36 | 23.11 | 33.958 | 78.33 | 12 |
| 9 | NDO-711 | 87.00 | 8 | 34 | 29.33 | 2.87 | 167.33 | 3038.33 | 6.75 | 28.56 | 31.37 | 94 | 13 |
| 10 | OL-1804 | 101.67 | 6.33 | 29 | 44.33 | 2.07 | 110. | 2026. | 4.53 | 22 | 41.07 | 97 | 15.44 |
| 11 | JHO-851 | 61.33 | 11.67 | 32.33 | 35.67 | 1.70 | 67 | 1314.67 | 2.92 | 26.11 | 29.45 | 91 | 11.33 |
| 12 | OS-377 | 93.33 | 10 | 37.67 | 39.67 | 2.00 | 110.67 | 2137.67 | 4.75 | 25.22 | 30.85 | 90 | 11 |
| 13 | PLP-1 | 71.67 | 8 | 36.67 | 39.33 | 2.20 | 102. | 2157 | 4.78 | 202.33 | 36.82 | 85.33 | 9.44 |
| 14 | NDO-1101 | 92.67 | 5 | 18.33 | 37 | 1.73 | 54.33 | 757.33 | 1.66 | 26 | 37.22 | 82 | 16 |
| 15 | HFO-114 | 79.33 | 10 | 38.67 | 45 | 1.60 | 89.33 | 1833.33 | 4.06 | 21.33 | 38.15 | 86 | 12.44 |
| 16 | NDO-1 | 92.00 | 5 | 25 | 46.67 | 2.30 | 82.67 | 1061 | 2.35 | 20.56 | 40.94 | 79.33 | 14.11 |
| 17 | KENT | 84.00 | 8 | 34 | 45.33 | 1.97 | 97 | 2051 | 4.55 | 21.11 | 36.95 | 80 | 11.44 |
| 18 | UPO-94 | 52.00 | 12 | 39.67 | 30.67 | 2.27 | 65.33 | 1908 | 4.17 | 20.44 | 32.23 | 85 | 9.56 |
| 19 | JHO-99-1 | 84.33 | 5 | 22 | 32.67 | 2.07 | 84.33 | 1021.33 | 2.26 | 23.67 | 28.10 | 84.33 | 9.78 |
| 20 | RO-19 | 105.33 | 8 | 43 | 45.33 | 2.80 | 170.33 | 3713.67 | 8.25 | 25.56 | 37.42 | 82.33 | 15.44 |
| 21 | NDO-2 | 110.33 | 7 | 28.33 | 40.67 | 2.00 | 103. | 1758 | 3.87 | 16.44 | 46.25 | 88 | 14 |
| 22 | OL-14 | 109.00 | 13.67 | 59.33 | 46 | 2.00 | 197.33 | 4156.67 | 9.23 | 11.44 | 30.60 | 88.67 | 6 |
| 23 | OL-1760/OL-11 | 80.67 | 10 | 52.33 | 43 | 1.93 | 166.33 | 3120 | 6.93 | 47.22 | 23.92 | 86.67 | 14.56 |
| 24 | OL-10 | 88.00 | 12.33 | 48.33 | 45 | 1.80 | 129.67 | 3311 | 7.35 | 30.89 | 37.07 | 91.33 | 18.56 |
| 25 | CSAOFC-14-4 | 104.33 | 8 | 32 | 50.67 | 2.73 | 128.67 | 2675.67 | 5.94 | 23.56 | 34.85 | 91.67 | 12.56 |
| 26 | JHO-2010-1 | 118.67 | 8 | 36.33 | 50.67 | 2.03 | 137.33 | 3136 | 6.96 | 29.33 | 34.08 | 87.67 | 15.33 |
| 27 | NDO-10 | 122.00 | 9 | 50 | 53 | 2.40 | 180 | 4346.33 | 9.65 | 45.44 | 24.36 | 86 | 14.44 |
| 28 | UPO-212 | 134.67 | 10.67 | 49.33 | 46.67 | 1.93 | 177.67 | 3052.33 | 6.78 | 42.89 | 27.68 | 84 | 16.44 |
| 29 | OL-1802 | 103.00 | 7.67 | 34.33 | 45 | 1.93 | 121.33 | 1952.67 | 4.34 | 20 | 35.72 | 82.33 | 11.56 |
| 30 | OL-1876-2 | 100.67 | 7 | 40.67 | 45.67 | 1.77 | 136.67 | 2152.33 | 4.78 | 27.67 | 25.10 | 88.33 | 9.44 |
| 31 | SKO-96 | 88 | 8.33 | 32.67 | 47 | 2.10 | 79 | 2193 | 4.87 | 32.56 | 31.46 | 87 | 15 |
| 32 | UPO-06-1 | 104.33 | 10.67 | 41 | 47 | 2.20 | 167.67 | 2978 | 6.68 | 20.11 | 31.46 | 90.33 | 9.44 |
| 33 | OL-1896 | 94 | 5 | 23 | 42 | 2.27 | 73.33 | 1256.33 | 2.79 | 23.11 | 28.57 | 92 | 9.44 |
| 34 | OS-405 | 110 | 8 | 38.33 | 43 | 2.43 | 142.33 | 2576.67 | 5.72 | 40 | 32.22 | 93.33 | 19 |
| 35 | RO-11-1 | 117.33 | 7.67 | 40.67 | 47 | 2.67 | 190 | 3345.67 | 8.54 | 50.67 | 17.56 | 95 | 10.56 |
| 36 | OS-6 | 94.67 | 9 | 41.67 | 38.33 | 1.27 | 108.33 | 1161 | 2.57 | 34.44 | 44.03 | 87 | 27.33 |
| 37 | OS-346 | 114.33 | 5.67 | 30. | 43.67 | 2.00 | 116 | 2050.67 | 4.56 | 41.44 | 38.23 | 93 | 25.44 |
| 38 | JHO-99-2 | 78 | 4 | 28.67 | 30 | 2.07 | 97 | 1142.33 | 2.53 | 26.56 | 25.20 | 97 | 9.50 |

| S.V | D.F. | Mean Sum of Square | | | | | | | | | | | |
| --- | --- | --- | --- | --- | --- | --- | --- | --- | --- | --- | --- | --- | --- |
|  |  | 1 | 2 | 3 | 4 | 5 | 6 | 7 | 8 | 9 | 10 | 11 | 12 |
| Replication | 2 | 41.12 | 1.48 | 18.97 | 6.64 | 0.12 | 38.75 | 22640.00 | 6.31 | 14.94 | 0.14 | 7.43 | 2.05 |
| Treatment | 37 | 888.60 | 17.32 | 265.38 | 118.48 | 0.43 | 4869.78 | 2815336.4 | 14.33 | 262.42 | 102.44 | 69.30 | 56.70 |
| Error | 74 | 2.72 | 1.22 | 17.91 | 1.23 | 1.09 | 92.64 | 19740.64 | 3.88 | 2.14 | 6.29 | 1.93 | 0.45 |

| Character | Mean | Range(Min-Max.) |
| --- | --- | --- |
| Plant Height(cm) | 95.1228 | 52.00-134.67 |
| Tillers per Plant | 7.85 | 4.00-13.67 |
| Leaf per Plant | 34.50 | 18.33-59.33 |
| Leaf Length( cm) | 41.48 | 30.00-53.00 |
| Leaf Width(cm) | 2.01 | 1.27-2.87 |
| Fresh Weight (g) | 114.78 | 56.33-197.33 |
| Leaf Area(cm^2^) | 2098.36 | 631.00-4346.33 |
| Leaf Area Index | 4.70 | 1.43-9.65 |
| Dry Weight (g.) | 29.19 | 20.00-50.67 |
| Harvest index (g) | 32.93 | 17.56-46.25 |
| Days of 50% Flowering | 87.69 | 79.33-97.00 |
| Grain Weight (g) | 14.05 | 6.00-27.33 |
