# Supplementary material for: Agro-morphological and genetic variability analysis in oat germplasms with special emphasis on food and feed
Source: PLoS One. 2023 Feb 8;18(2):e0280450. doi: 10.1371/journal.pone.0280450 (PMC9907803; doi:10.1371/journal.pone.0280450)

Fig.1- PCR amplification profile of 38 oat germplasms using UBC 826 Inter Simple Sequence Repeats (ISSRs) markers along with ladder

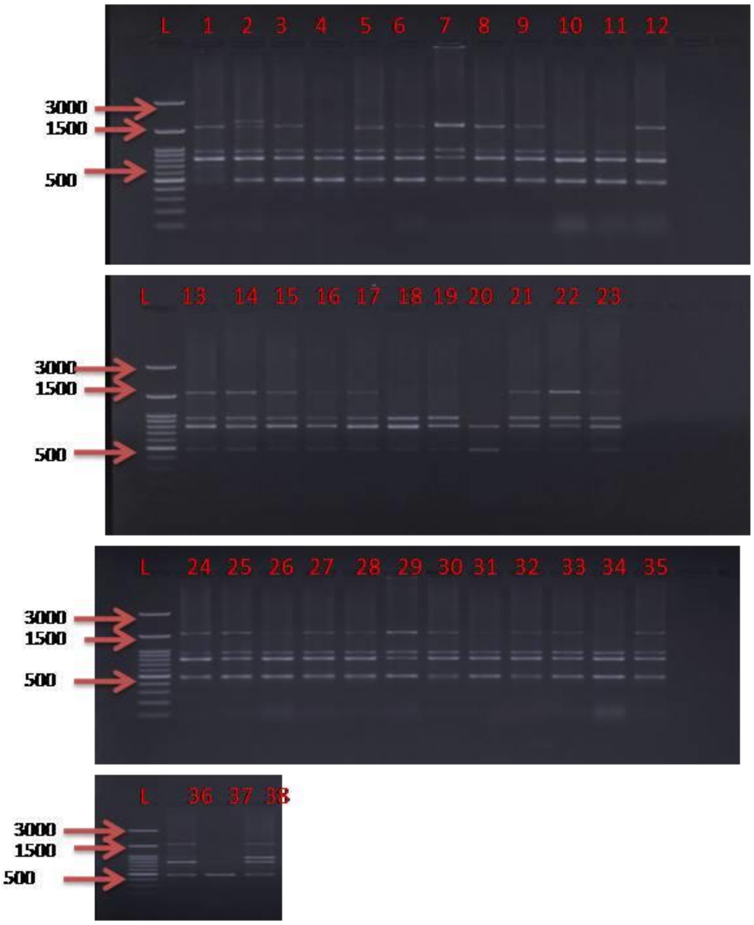

Fig.2- Dendrogram of 38 *Avena sativa L.* based on 22 Inter Simple Sequence Repeats (ISSRs) markers

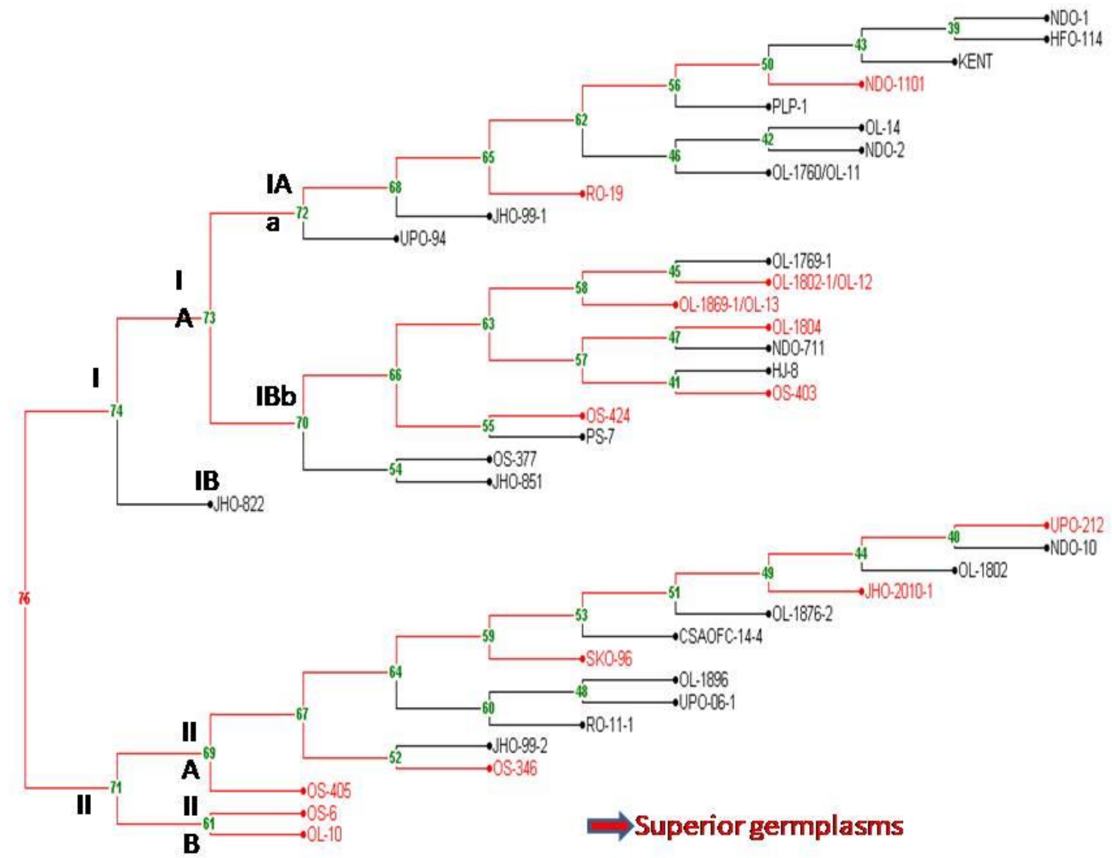

Fig.3-Inter Simple Sequence Repeats (SSR) marker-based principal component analysis showing two-dimensional distributions of oat germplasms

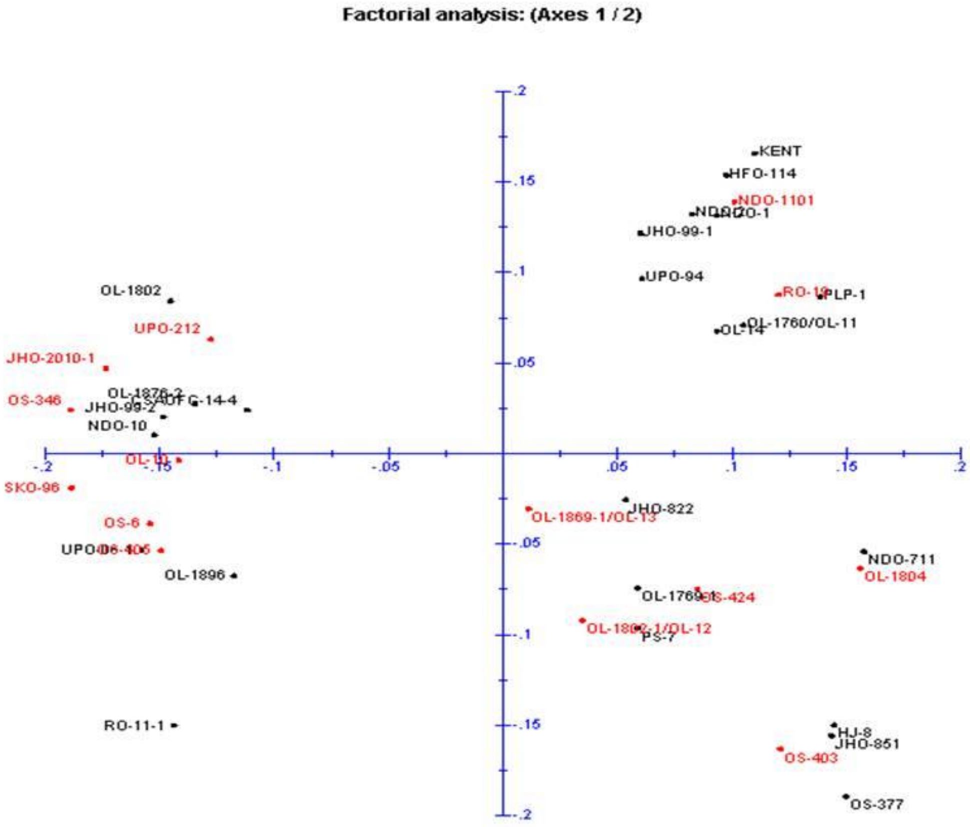

Fig.4- Bayesian model-based genetic clustering of 38 oat germplasms

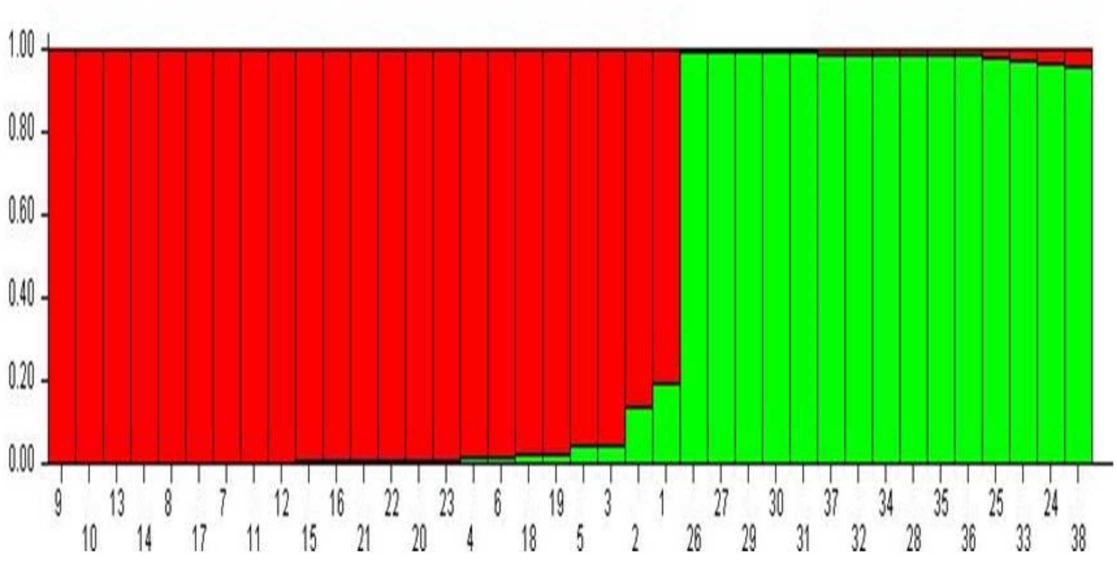

Supplementary Fig. 1a- Heat map depicting grain yield profile of 38 oat germplasms

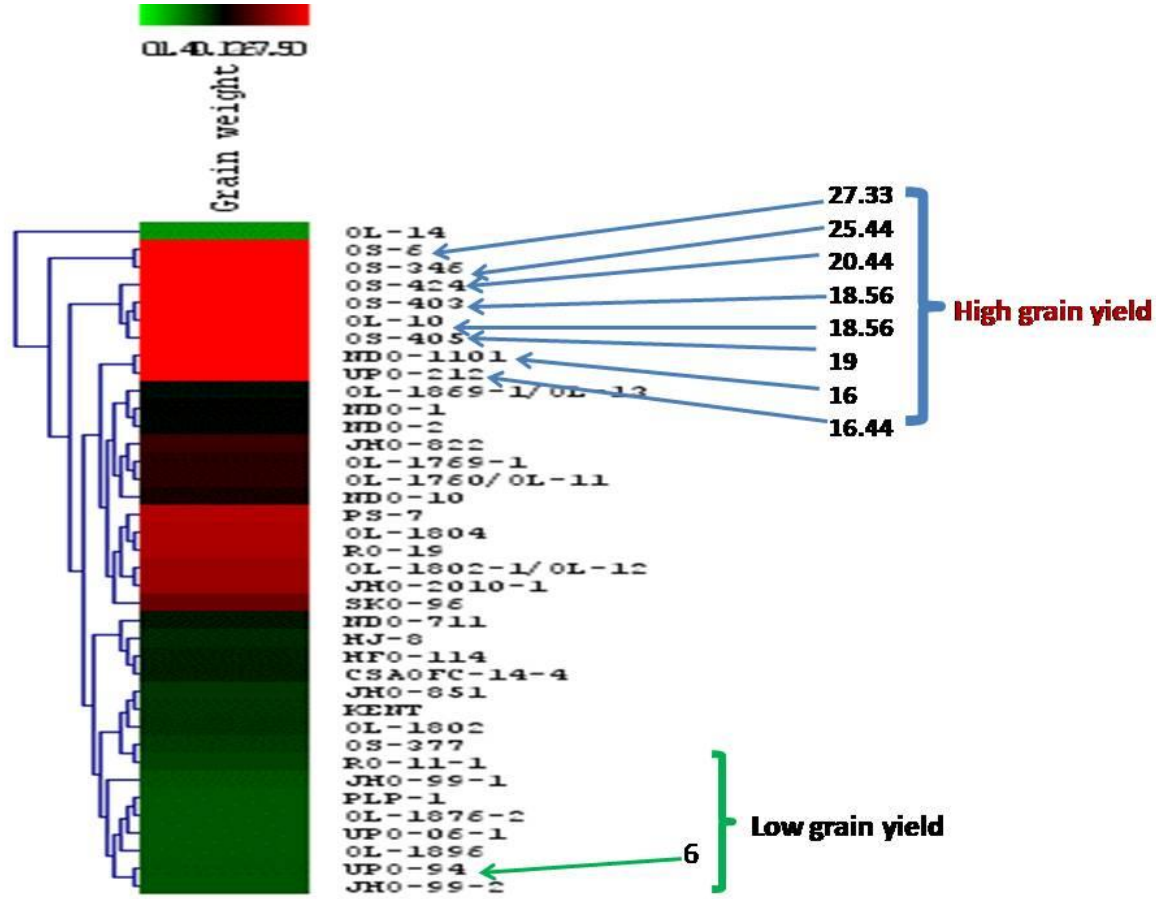

Supplementary Fig. 1b-Correlation heatmap matrix of grain yield profile of 38 oat germplasms

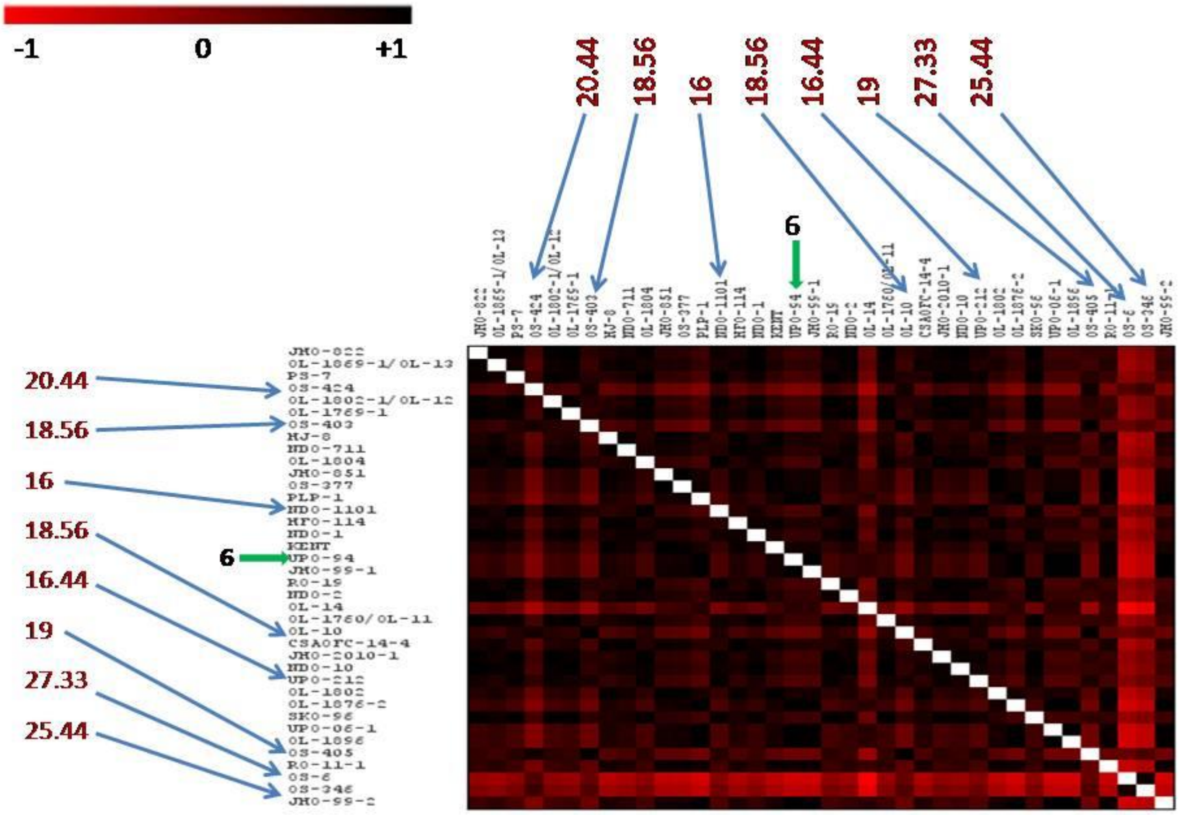

Supplementary Fig.2a-Heat map depicting fresh weight (for fodder purpose) profile of 38 oat germplasms

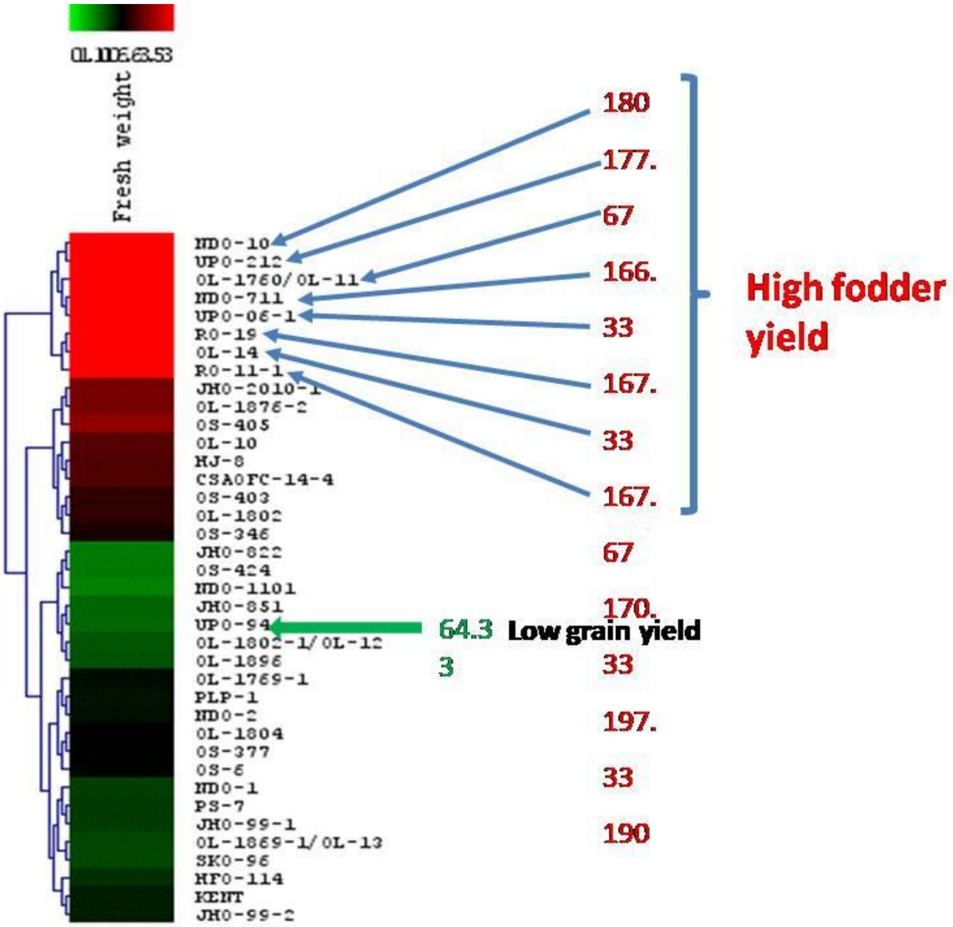

Supplementary Fig. 2b- Correlation heatmap matrix of fresh weight (for fodder purpose) profile of 38 oat germplasms

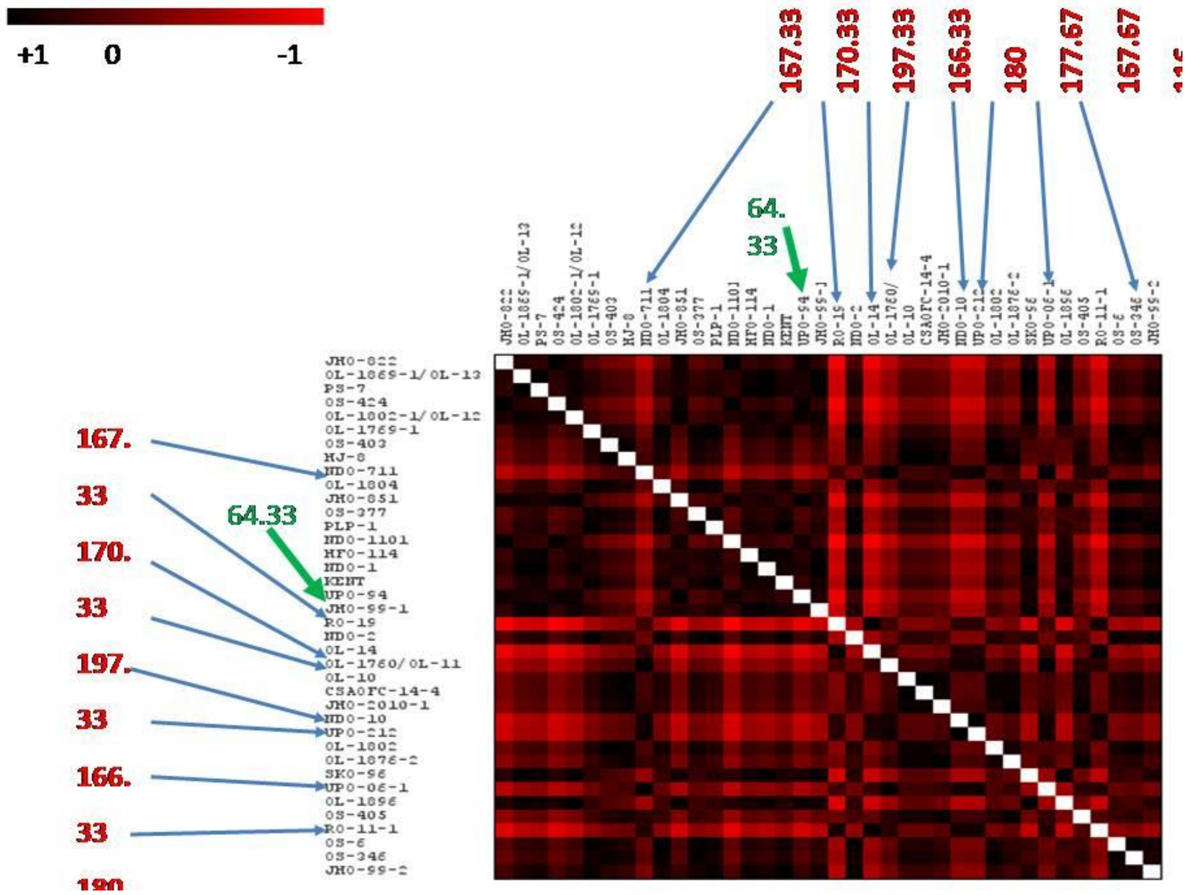

Supplement: S1 Raw images — (PDF) [file pone.0280450.s006.pdf]
