# Supplementary material for: Agro-morphological and genetic variability analysis in oat germplasms with special emphasis on food and feed
Source: PLoS One. 2023 Feb 8;18(2):e0280450. doi: 10.1371/journal.pone.0280450 (PMC9907803; doi:10.1371/journal.pone.0280450)
Supplement: Attachment — Submitted filename: Response to Reviewers.docx [file pone.0280450.s007.docx]

To,

The Editor

PLOS ONE

Please find enclosed our detailed responses to the reviewers’ and editor’s comments for the manuscript “Agro-morphological variability, population structure, and genetic diversity in oat germplasms with emphasis on food and feed.” The Editor’s and reviewer’s comments were very constructive and mainly concerned (1) Overall writing of the MS, (2) Novelty of the work and (3) Overall statistical analysis, discussion and conclusion. We appreciate the time and effort that you and the reviewers dedicated to providing feedback on our manuscript. We have dealt with all of the critical comments in full, and thoroughly revised the manuscript. We hope that you agree that we have satisfactorily dealt with all of the reviewer’s comments in full.

Sincerely,

Deepanshu Jayaswal on behalf of all authors.

**Response to Reviewer 1**

**Comment 1:** Abstract does not properly explain the importance of study, material and methods, and results. It is confusing and difficult to understand. All the required information should be added according to the journal format.

**Response:** Thanks for your valuable comments. We have gone through the manuscript and rewrote the abstract keeping the points as suggested.

**Comment 2:** Introduction does not provide the proper background of study and objectives. Also does not illustrate the importance of the method and why it was chosen for this study.

**Response:** In the introduction part, reference number 9, 10, 11, 12, 13, and 14 have been cited to support the statements regarding application of molecular markers for molecular breeding. The objective of the study is to identify the relatedness between selected germplasm so that after studying genetic diversity, the superior performing germplasm could be used for molecular breeding to improve the food and feed quality with higher yield.

The importance of the method is by using ISSR molecular marker; we can exploit the multiple alleles linked with the traits. ISSR markers are dominant in nature; hence, phenotypes can be differentiated at each locus in the Mendelian pattern. It is chosen because of their advantage over other markers (Line number 82-88).

**Comment 3:** Material and Method, does not provide information about the origin of the material. Does not explain how morphological traits were measured and what different softwares were used for analysis. The annealing temperature given for primers is not correct. All experimental conditions should be clearly explained.

**Response:** The seed material has been collected from Universities have been provided in Supplementary table 2. The morphological traits were taken on field crops in triplicates. **Days to 50 % flowering**: the days were recorded when 50 % flowering from the date of showing has been completed. **Plant height:** The plant height was measured when plants became matured and further growth was negligible. Rest of the parameters were observed in the respective manner of the concerned traits. Due to make the material and method short and crispy, we did not explain about how to measure all the traits because the terms used are self-explanatory in most of the cases. Statistical analysis were performed on excel and Mev software for heatmap was used (Included in material and method).

The annealing temperature was optimised by gradient PCR. Because of single primer (ISSR), at 50 **^o^C,** all **the** primers were amplified well; hence the common annealing temperature was used for all the primers. For detail PCR conditions, we have cited the research article from our lab Yadav et al., 2022.

**Comment 4:** Result and discussion: Results are not properly explained, important information was skipped. All characters should be explained properly and it should be explained what parameters were chosen for selection as fodder or food. Also, include at what stage data of specific trait was measured. All findings should be properly discussed with relevant support from the literature and their significance to the scientific community and breeders.
Figure legends are not comprehensive, should be written properly with the required information.
The author's contribution is not mentioned.

**Response:** We have gone through the results and explained as per data. The yield of the grain per plant in gram was taken for food and high fresh weight was taken for fodder (line number 105-106). The stage at which data were taken has been included as per suggestions (line number 106-107). Figure legends have been rectified as per need. The authors contribution have been provided during submission of the MS.

**Comment 5:**

References are not properly formatted and need to be corrected properly.
Language: The language is poor and needs a thorough revision with proper sentence structure. Do not use multiple words for a single thing e.g. accessions, lines, genotypes. Use a single word either accessions or lines. It should be followed throughout the manuscript.
Formatting: The paper should be properly formatted as per journal requirements.
After making all major revisions the manuscript should be submitted.

**Response:** All suggested comments have been considered and rectified where needed.

**Response to Reviewer 2**

**Comment 1:**	Scientific novelty of your manuscript is not clear. The data of your manuscript are important for local breeders and farmers. International novelty of your manuscript seems to	be	low.

**Response:** While we appreciate reviewers’ feedback, we respectfully disagree with the reviewer in their assessment regarding novelty. The used germplasms were unique and ISSR markers used were sowing high allelic variability. This comment is also in contrast with those of the journal requirement ‘**’evaluate submitted manuscripts on the basis of methodological rigor and high ethical standards, regardless of perceived novelty’’**.

**Comment 2:** Your experiments were carried out in one season with three replications. The weather condition can influence significantly the data of your experiments. There is no information about the field trials and the germplasm.

**Response:** Yes, we are agreeing with the reviewer’s valuable comment that the phenotypic data were taken in one season and it can be influenced by environmental conditions. Therefore, classifying the oat germplasms for desirable traits based on phenotypic data is not reliable. Keeping these things in mind, we have genotyped the oat germplasms with molecular marker ISSRs, so that after looking the dendrogram, PCA and structure analysis, we found negligible difference in the classification by both ways.

The information about germplasm has been given in supplementary table S1. The field trials have been given in material method section **Analysis of agronomic morphological and yield traits.**

**Comment 3:** Whether the germplasm have novelty?

**Response:** Yes, few germplasm have some unique traits that are not available in others. Hence, they can be used for allele mining that is agriculturally important.

**Comment 4:** The marker density is too low as 22 markers used in the study. The relationship between marker data and agronomic traits is not clear.

**Response:** Yes, we agree that marker density is too low. The reason behind this is the ability of ISSR markers. ISSRs markers exploit more allelic difference in comparison to other markers. In our case, the allelic range was 6-12 in number and the size of alleles were 350-1900. On the other hand, markers like SSRs exploit only few alleles to see the difference between organisms.

Basically in the current study, phenotypic data from field have been observed and recorded. Based on the selected phenotypic data (Grain yield and Fresh leaf weight), we have classified the germplasm. Further to validate the classification, genotypic data was analysed by dendrogram, Principal component analysis and structure.

**Comment 5:** In the ‘Discussion’, you should enhance the novelty of your experiments in comparison with the most relevant international publications.

**Response:** Thanks for invaluable suggestion. We have gone through the discussion part too and rectified as per suggestion.
