# Supplementary material for: Agro-morphological and genetic variability analysis in oat germplasms with special emphasis on food and feed
Source: PLoS One. 2023 Feb 8;18(2):e0280450. doi: 10.1371/journal.pone.0280450 (PMC9907803; doi:10.1371/journal.pone.0280450)
Supplement: Attachment — Submitted filename: PONE-D-2226734 - Comments.doc [file pone.0280450.s008.doc]

Dear Editor

I have gone through paper entitled ***“Agro-morphological variability, population structure, and genetic diversity in oat germplasm with special emphasis on food and feed”***.

Although, the several studies have been published on the same topic, nothing new in this paper. However, the paper is generally well organized, and the introduction gave a literature review of related topics, and summarized the methods proposed in this paper. In order to make the paper easy to understand, appropriate figures and tables were also given. Therefore, I think that the paper could be published in the Journal with some modifications.

Here my comments and suggestions:

- Still some grammatical errors are present, that should be removed before publication.
- Title should be more appropriate
- Key words are not appropriate
- Write full forms of all abbreviations at least once.
- Sampling and data recording is clear and significant.
- All Figures are poor and not clear, should be replace with clear and readable images.
- Also check all references in the text and reference list carefully some are missing from the list or not cited in the text.
- Most importantly, the discussion section needs to be strengthened to highlight the importance of the work.

**For further justification of the study, kindly incorporate the below current review, please.**

Al-Yasari MNH (2022). [Potassium and nano-copper fertilization effects on morphological and production traits of oat (*Avena sativa* L.)](https://sabraojournal.org/potassium-and-nano-copper-fertilization-effects-on-morphological-and-production-traits-of-oat-avena-sativa-l/). SABRAO J. Breed. Genet. 54(3): 678-685. http://doi.org/10.54910/sabrao2022.54.3.20.

Abdel-Lateif KS, Hewedy OA (2018). Genetic diversity among Egyptian wheat cultivars using SCoT and ISSR markers. *SABRAO J. Breed. Genet.* 50: 36–45.

Qulmamatova DE, Baboev SK, Buronov AK (2022). [Genetic variability and inheritance pattern of yield components through diallel analysis in spring wheat](http://sabraojournal.org/genetic-variability-and-inheritance-pattern-of-yield-components-through-diallel-analysis-in-spring-wheat/). *SABRAO J. Breed. Genet.* 54(1): 21-29. http://doi.org/10.54910/sabrao2022.54.1.3

Helsel DG, Skrdla RK (1983). Breeding for grain yield in oats (*Avena sativa* L.). SABRAO J. Breed. Genet. 15(2): 139-145.

Swailam MA, Mowafy SAE, El-Naggar NZA, Mansour E (2021). [Agronomic responses of diverse bread wheat genotypes to phosphorus levels and nitrogen forms in a semiarid environmen](http://sabraojournal.org/?p=1449)t. *SABRAO J. Breed. Genet.* 53: 592-608. https://doi.org/10.54910/sabrao2021.53.4.4.
