# Supplementary material for: Agro-morphological and genetic variability analysis in oat germplasms with special emphasis on food and feed
Source: PLoS One. 2023 Feb 8;18(2):e0280450. doi: 10.1371/journal.pone.0280450 (PMC9907803; doi:10.1371/journal.pone.0280450)
Supplement: Attachment — Submitted filename: Response to Reviewers.docx [file pone.0280450.s009.docx]

To,

The Editor

PLOS ONE

Please find enclosed our detailed responses to the reviewers’ and editor’s comments for the manuscript “**Agro-morphological and genetic variability analysis in oat germplasms with special emphasis on food and feed**”. The Editor’s and reviewer’s comments were very constructive and mainly concerned (1) Format, title and pictures quality MS, (2) References and the grammar. We appreciate the time and effort that you and the reviewers dedicated to providing feedback on our manuscript. We have dealt with all of the critical comments in full, and revised the manuscript. We hope that you agree that we have satisfactorily dealt with the reviewer’s comments in full.

Sincerely,

Deepanshu Jayaswal on behalf of all authors.

**Response to Editor**

**Comment 1:** A rebuttal letter that responds to each point rose by the academic editor and reviewer(s). You should upload this letter as a separate file labeled 'Response to Reviewers'.

**Response:** The Response to Reviewers file has been attached.

**Comment 2:** A marked-up copy of your manuscript that highlights changes made to the original version. You should upload this as a separate file labeled 'Revised Manuscript with Track Changes'.

**Response:** 'Revised Manuscript with Track Changes' with suggested comments has been attached.

**Comment 3:** An unmarked version of your revised paper without tracked changes. You should upload this as a separate file labeled 'Manuscript'.

**Response:** Submitted as suggested.

**Response to Journal Requirements**

**Comment 1:** Please ensure that your manuscript meets PLOS ONE's style requirements, including those for file naming.

**Response:** The whole manuscript has been considered as per the journal requirement and completed as per requirement.

**Comment 2:** In your Data Availability statement, you have not specified where the minimal data set underlying the results described in your manuscript can be found. PLOS defines a study's minimal data set as the underlying data used to reach the conclusions drawn in the manuscript and any additional data required to replicate the reported study findings in their entirety. All PLOS journals require that the minimal data set be made fully available**.**

**Response:** The minimal data requirements have been provided in all the attached supplementary tables.

**Comment 3:** PLOS ONE now requires that authors provide the original uncropped and unadjusted images underlying all blot or gel results reported in a submission’s figures or Supporting Information files**.**

**Response:** We would like to ask for an apology for this. The student has taken the gel images and cropped without saving the original images. Therefore, we have all the images but in cropped version. For this we are in regret and can not produce the uncropped image. Meantime we will try to recover the images from the gel doc system where the images were taken.

**Comment 4:** Please include captions for your Supporting Information files at the end of your manuscript, and update any in-text citations to match accordingly.

**Response:** Legends captions for all the figures and tables are given after references.

**Comment 5:** Please review your reference list to ensure that it is complete and correct. If you have cited papers that have been retracted, please include the rationale for doing so in the manuscript text, or remove these references and replace them with relevant current references. Any changes to the reference list should be mentioned in the rebuttal letter that accompanies your revised manuscript. If you need to cite a retracted article, indicate the article’s retracted status in the References list and also include a citation and full reference for the retraction notice.

**Response:** We have gone through the MS and checked for all references.

**Response to Reviewers**

**Comment 1:** Still some grammatical errors are present, that should be removed before publication.

**Response:** The authentic purchased software was used to rectify the grammatical mistekes.

**Comment 1:** Title should be more appropriate

**Response:** As the term diversity itself includes population structure analysis therefore, title has been modified to make it bit short. **Agro-morphological and genetic variability analysis in oat germplasms with special emphasis on food and feed**

**Comment 2:** Key words are not appropriate

**Response:** Keywords has been replaced by those which are involved many times in the MS and represent the research work.

**Comment 3:** Write full forms of all abbreviations at least once.

Response: Please accept our apology for such small but key mistakes. Now, we have corrected as per suggestion.

**Comment 4:** Sampling and data recording is clear and significant.

**Response:** We are highly grateful for appreciation.

**Comment 5:** All Figures are poor and not clear, should be replacing with clear and readable images.

**Response**: The entire pictures dpi has been upgraded up to 600 dpi to enhance the visibility.

**Comment 6:** Also check all references in the text and reference list carefully some are missing from the list or not cited in the text.

**Response**: We have carefully checked the total number of references given and their citations in the MS text and rectified as per necessity.

**Comment 7:** Most importantly, the discussion section needs to be strengthened to highlight the importance of the work.

**Response:** Discussion of each finding has been explained where needed as per suggestion.
